# Skeletal muscle mass, muscle strength, and physical performance in children and adolescents with obesity

**DOI:** 10.3389/fendo.2023.1252853

**Published:** 2023-10-04

**Authors:** Marcela Zembura, Kamila Czepczor-Bernat, Patrycja Dolibog, Paweł T. Dolibog, Paweł Matusik

**Affiliations:** ^1^ Department of Pediatrics, Pediatric Obesity and Metabolic Bone Diseases, Faculty of Medical Sciences in Katowice, Medical University of Silesia, Katowice, Poland; ^2^ Department of Medical Biophysics, Medical University of Silesia, Katowice, Poland; ^3^ Department of Biophysics, Faculty of Medical Sciences, Medical University of Silesia, Zabrze, Poland

**Keywords:** sarcopenic obesity, sarcopenia, childhood obesity, muscle mass, muscle strength, physical performance

## Abstract

**Introduction:**

Sarcopenic obesity (SO) is defined as obesity with low skeletal muscle function and mass. This study aimed to evaluate the presence of sarcopenic obesity according to different diagnostic criteria and assess the elements of sarcopenia in children and adolescents with obesity.

**Methods:**

A total of 95 children and adolescents with obesity (diagnosed with the use of International Obesity Task Force (IOTF) criteria) with a mean age of 12.7( ± 3) years participated in the study. Body composition was assessed with the use of bioelectrical impedance—BIA (Tanita BC480MA) and dual-energy X-ray absorptiometry—DXA (Hologic). Fat mass (FM) and appendicular skeletal muscle mass (SMMa) were expressed as kilograms (kg) and percentage (%). Muscle-to-fat ratio (MFR) was defined as SMMa divided by FM. A dynamometer was used in order to measure grip strength. Six-minute walk test (6MWT) and a timed up-and-go test (TUG) were used to assess physical performance.

**Results:**

The presence of SO ranged from 6.32% to 97.89%, depending on the criteria used to define sarcopenia. Children with sarcopenia, defined as a co- occurrence of low skeletal muscle mass % (SMM%) measured by DXA (≤9th centile) according to McCarthy et al. and weak handgrip strength (≤10th centile) according to Dodds et al., had significantly lower SMMa measured by both DXA and BIA, lower maximal handgrip strength, and lower physical performance. Maximal handgrip was positively correlated with SMMa (kg) and SMMa% derived from both DXA and BIA and BIA-MFR. Maximal handgrip was negatively correlated with waist-to-height ratio (WHtR). The distance of 6MWT correlated positively with BIA-measured SMMa% and BIA-MFR. 6MWT distance correlated negatively with BIA-FM% and body mass index (BMI) z-score. TUG was positively correlated with BIA-FM%, BMI z-score, WHtR, and IOTF categories and negatively correlated with BIA-SMMa% and BIA-MFR.

**Discussion:**

The presence of sarcopenia in our study varied depending on the diagnostic criteria used. This is one of the first studies evaluating muscle mass, muscle strength, and physical performance in children and adolescents with obesity. The study highlighted the need for the implementation of a consensus statement regarding SO diagnostic criteria in children and adolescents.

## 1 Introduction

The rising prevalence of obesity in the pediatric population is a growing problem for public health. Over 340 million children and adolescents aged 5–19 were overweight or obese in 2016, according to the data from the World Health Organization (WHO) (1). The WHO European Regional Obesity Report 2022 indicated that nearly one in three children (29% of boys and 27% of girls) is affected by overweight and obesity in the WHO European Region. Various studies from the European Region found that overweight and obesity prevalence and/or mean body mass index increased during the coronavirus disease 2019 pandemic in children and adolescents (2). According to the World Obesity Atlas 2022, which evaluated obesity prevalence among children and adolescents regarding the income level of the countries in 2020, the highest prevalence of obesity in children and adolescents was found in middle-income countries. The projections show that over 80 million children aged 5–9 and over 110 million children aged 10–19 from middle-income countries are going to be affected by obesity by the year 2030 (3). Various comorbidities such as hyperinsulinemia, insulin resistance, type 2 diabetes mellitus, polycystic ovarian syndrome, obstructive sleep apnea, nonalcoholic fatty liver disease, elevated blood pressure, musculoskeletal problems, and psychosocial consequences such as poor self-esteem, anxiety, and depression are associated with obesity (4). A strong correlation was found between obesity in childhood and adulthood, as children and adolescents with obesity are five times more likely to become obese adults (5). Obesity in childhood was found to be a cardiovascular disease risk factor and might be associated with early atherosclerosis and premature cardiovascular disease in adulthood (5).

Sarcopenia was first described in 1989 by Irwin Rosenberg, who proposed the term “sarcopenia” (Greek “sarx” or flesh + “penia” or loss) to describe this age-related decrease of muscle mass (6, 7). In 2019, a revised consensus regarding sarcopenia was published by the European Working Group on Sarcopenia in Older People (EWGSOP2) (8). According to the EWGSOP2, sarcopenia could be diagnosed when low muscle strength is present, and diagnosis is confirmed when low muscle mass occurs, additionally, if low physical performance is present, sarcopenia is considered severe.

Sarcopenic obesity (SO) is defined as obesity with low skeletal muscle function and mass (9). Sarcopenia and obesity are both parts of a vicious cycle since sarcopenia leads to a reduction of physical activity, which leads to decreased energy expenditure and increased risk of obesity, and increased visceral fat results in inflammation, which impacts the development of sarcopenia (10, 11). Both sarcopenia and obesity are associated with adverse health outcomes; therefore, the co-occurrence of both conditions may synergistically amplify the health risks (12). According to the European Society for Clinical Nutrition and Metabolism and European Association for the Study of Obesity Consensus Statement regarding sarcopenic obesity in adults, SO can be diagnosed if both altered skeletal muscle functional parameters considering strength (hand-grip strength, chair stand test) and altered body composition—increased fat mass (FM) and reduced muscle mass assessed by appendicular lean mass adjusted to body weight by dual X-ray absorptiometry (DXA) or as skeletal muscle mass adjusted by weight by bioelectrical impedance analysis (BIA)—are present (13).

Primarily, SO was considered to be a condition associated with the elderly population, considering age-related changes in body composition, hormone levels, and proinflammatory pathways (12). However, SO is now also linked to the pediatric population, as results of pediatric studies show a significant association between low skeletal muscle mass (SMM), obesity, and adverse health outcomes in children and adolescents (14–19).

However, the lack of a consensus statement regarding SO diagnosis, diagnostic methods, and gender-, age-, and race-specific cut-off points in the pediatric population hampers the progress of the research in this field (20).

To our knowledge, this is one of the first studies evaluating all of the components of sarcopenia, namely muscle mass, muscle strength, and physical performance, in children and adolescents with obesity.

This study aimed to evaluate the presence of sarcopenic obesity according to different diagnostic criteria and assess the components of sarcopenia (muscle mass, muscle strength, and physical performance) in children and adolescents with obesity.

## 2 Materials and methods

### 2.1 Study group description

The study comprised 95 Caucasian children and adolescents with obesity, with a mean age of 12.7 ( ± 3). Obesity was diagnosed according to International Obesity Taskforce (IOTF) criteria, which provide international child cut-offs corresponding to the body mass index (BMI) cut-offs at 18 years (27 for obesity grade 1, 30 for obesity grade 2, and 35 for obesity grade 3–morbid obesity). Cut-offs are provided for exact ages by month, from 2 years to 18 years (21). Children in the age range 7–18 years were included. Patients were recruited consecutively during their hospitalization in our clinic, between February 2022 and August 2022.

Exclusion criteria included children with monogenic disorders and genetic syndromes associated with obesity, endocrine disorders resulting in obesity, hypothalamic causes of obesity, drug-induced obesity, and neuromuscular disorders affecting muscle quality and quantity.

#### 2.1.1 Anthropometric measurements

Standing height was measured to the nearest 0.1 cm with the use of a wall-mounted stadiometer. Weight was measured without shoes and in light clothes to the nearest 0.1 kg. BMI was defined as weight divided by height squared (kg/m^2^). BMI was converted to age- and gender-specific *z*-scores using WHO AnthroPlus, which is the global application of the WHO Reference (22). The BMI *z*-score is expressed as a number of standard deviations (SD) from the value of the 50th percentile (median). The WHO Growth Reference Data from 2007 were used. The waist circumference was measured with flexible tape midway between the lower rib margin and the iliac crest in the standing position, and the waist-to-height ratio (WHtR) was calculated.

#### 2.1.2 Body composition analysis

Body composition was evaluated using BIA (Tanita BC480MA) and DXA (Hologic). BIA provided values of fat-free mass (FFM) and FM and predicted skeletal muscle mass in four extremities. DXA measured bone mineral content (BMC; g), bone mineral density (BMD; g/cm^2^), FM (g), and FFM including BMC (g). Appendicular muscle mass (SMMa; kg) was calculated as the sum of the predicted muscle mass of upper and lower limbs in BIA and as the sum of the lean soft tissue masses in legs and arms; regarding that, all nonfat and nonbone tissues are skeletal muscle in DXA. SMMa% was defined as SMMa/weight. FM% was defined as FM/weight. The muscle-to-fat ratio (MFR) was calculated according to McCarthy et al. by dividing SMMa (kg) by FM (kg) (23).

#### 2.1.3 Muscle strength assessment

With the use of the Saehan dynamometer, grip strength was measured for both hands. Grip strength was evaluated according to the American Society of Hand Therapists recommendations (24). Therefore, three consecutive trials were carried out for both the dominant and nondominant hands and recorded in kilograms with the second handle position of the dynamometer. The patient was seated with his shoulder adducted and neutrally rotated, elbow flexed at 90°, and the forearm and wrist in neutral position with the examiner’s light support of the base of the instrument. The best value from all six trials was used as the maximal handgrip strength. Values ≤10th centile for gender and sex, according to Dodds et al., were considered to have weak handgrip strength (25).

#### 2.1.4 Physical performance analysis

The 6-min walk test (6MWT) was performed on all of the patients. 6MWT was conducted indoors, along a 30-m-long corridor, with the use of a measuring wheel. The handling bar of the measuring wheel was adjusted to the children’s height. Patients wore light clothes and shoes appropriate for walking and were told not to exercise vigorously 2 h before the test. Blood pressure, heart rate, and pulse oximetry were measured before and right after the test. Patients were instructed to “Walk as far as possible for 6 min. You will walk back and forth in this hallway. Six minutes is a long time to walk, so you will be exerting yourself. You will probably get out of breath or become exhausted. You are permitted to slow down, to stop, and to rest as necessary. You may lean against the wall while resting, but resume walking as soon as you are able. Are you ready to do that? Remember that the object is to walk AS FAR AS POSSIBLE for 6 min, but don’t run or jog. Start now, or whenever you are ready” according to the American Thoracic Society (ATS) Statement (26). After each minute, the participant was told in an even tone the following: “You are doing well. You have 5 min to go.”, “Keep up the good work. You have 4 min to go.”, “You are doing well. You are halfway done.”, Keep up the good work. You have only 2 min left.”, and “You are doing well. You have only 1 min to go.” according to ATS Statement (26). After 6 min, the participant was told to stop, and the distance from the measuring wheel was recorded.

A timed up-and-go test (TUG) was performed on all of the participants. A demonstration of the test task was performed prior to the test. Individuals were instructed to sit in the armchair with a backrest and without an armrest and to walk as fast as they could during the test. The time needed to stand up from a seated position in a chair, walk 3 m, turn around, return to the chair, and sit down was measured.

#### 2.1.5 Sarcopenia assessment

Three different diagnostic criteria were used to define sarcopenia: co-occurrence of weak handgrip strength (≤10th centile) according to Dodds et al. (25) and low skeletal muscle mass % (SMM%) (≤2nd centile) according to McCarthy et al. (23); co-occurrence of weak handgrip strength (≤10th centile) according to Dodds et al. (25) and low SMM%(≤9th centile) according to McCarthy et al. (23); and MFR cut-off value according to McCarthy et al. (23) (cut off = mean value −2 SD of the MFR of the middle fifth of the BMI range): 1.25 for boys all ages, 1.1 for girls between 5 and 10 years, and 0.8 for girls between 10 and 18 years. All of the diagnostic criteria mentioned above were used for both BIA- and DXA-derived measurements. It should be noted that diagnostic criteria, including both handgrip strength and low SMM%, were introduced by the authors for the first time. The authors used normative values for grip strength, stratified by gender by Dodds et al. (25), and decided to use the lowest centile (≤10th) available in the normative values for grip strength as a cut-off point. Tabulated % skeletal muscle mass centile values by exact age by McCarthy et al. (23) were also used, and the lowest centiles (≤2nd centile, ≤9th centile) available in the tabulated %skeletal muscle mass centile values were used. Moreover, the authors used the MFR cut-off value according to McCarthy et al. (23), since according to the systematic review by Zembura et al. (20), mean MFR-2SD of the third BMI quintile was the most prevalent definition of SO used in the studies included in the systematic review regarding sarcopenic obesity in children and adolescents.

Only the assessment of both muscle strength and muscle mass is in line with the EWGSOP2 consensus; therefore, a definition of sarcopenia consisting of both muscle strength and mass was used in the further part of the study. The authors decided to use SMM% values in the ≤9th centile as a cut-off point for further analysis to avoid inconsistency (since values ≤10th centile were used as a cut-off point for grip strength).

### 2.2 Statistical analysis

At the beginning of the study, the descriptive statistics of the study population were calculated. Data were presented as mean ± SD for continuous variables normally distributed, median (upper quartile and lower quartile) for continuous variables non-normally distributed, and percentage for categorical variables.

The analysis included two problems. The first was the comparison of studied variables in different groups of patients. The second issue was related to the analysis of relationships between two variables.

The procedure of variables comparison included several stages. The first normal distribution of the data was checked with the Shapiro–Wilk test. Next, variance homogeneity was tested with Leven’s or Bartlett’s tests. Normally distributed parameters with variance homogeneity were tested with a *t*-test. Otherwise, Welch’s *t*-test was used. Parameters without normal distribution were tested using the Mann–Whitney *U*-test.

The Chi-squared test of independence was used to determine whether there is an association between categorical variables. The relationship between continuous variables was analyzed using partial correlation coefficients to exclude the effect of gender and age. The test for conditional independence of two variables given the other ones was used to check the statistical significance of the obtained partial correlation coefficients. All calculations were performed in the R language with the ppcor package.

It was assumed for all tests that a *p*-value of < 0.05 indicates that the results are statistically significant. The analysis was conducted with the use of Microsoft Excel and the R computing system.

### 2.3 Ethical considerations

The study was carried out with approval from the faculty’s ethics committee (Approval No. PCN/CBN/0022/KB1/129/I/21/22). Written informed consent was obtained from parents or legal guardians of all children prior to their participation in the study.

## 3 Results

### 3.1 Characteristics of the study population

Female and male patients differed significantly in terms of age and BMI *z*-score. There were no significant differences regarding WHtR and severity of obesity according to IOTF criteria—international child cut-offs corresponding to the BMI cut-offs at 18 years (27 for obesity grade 1, 30 for obesity grade 2, and 35 for obesity grade 3–morbid obesity) (21). Regarding body composition assessment with the use of BIA and DXA, girls had significantly higher FM assessed by both BIA and DXA and FM% evaluated by BIA. Boys had significantly higher SMMa% assessed by both BIA and DXA, BIA-MFR, and a distance of 6MWT (**Table 1**).

**Table 1:** Characteristics of the study population.

*n*	Overall	Girls	Boys	*p*-value
	95	43	52	NA
Age	12.5 (10.08; 15.5)^a^	13.6 (11.5; 16.2)^a^	11.4 (9.73; 13.4)^a^	**<0.01**
BMI *z*-score	2.91 (2.57; 3.5)^a^	2.73 (2.48; 3.08)^a^	3 (2.71; 3.65)^a^	**<0.01**
WHtR	0.61 (0.56; 0.64)^a^	0.59 (0.54; 0.63)^a^	0.61 (0.59; 0.64)^a^	0.28
IOTF	**(1)** 33.68%^c^ (*n* = 32)	**(1)** 37.21%^c^ (*n* = 16)	**(1)** 30.76%^c^ (*n* = 16)	0.8
	**(2)** 32.64% (*n* = 31)	**(2)** 30.23% (*n* = 13)	**(2)** 34.62% (*n* = 18)	
	**(3)** 33.68% (*n* = 32)	**(3)** 32.56% (*n* = 14)	**(3)** 34.62% (*n* = 18)	
BIA				
FM (kg)	30 (22.8; 40.1)^a^	32.8 (25; 47)^a^	27.4 (22.2; 33.8)^a^	**<0.05**
FM%	38.59 ( ± 6.15)^b^	40.1 ( ± 5.74)^b^	37.4( ± 6.27)^b^	**<0.05**
SMMa (kg)	21.3 (16.1; 24.55)^a^	21.5 (17.7; 24.4)^a^	19.6 (15.8; 27.2)^a^	0.99
SMMa%	26.52 ( ± 2.6)^b^	24.6 ( ± 1.55)^b^	28.1 ( ± 2.17)^b^	**<0.001**
MFR	0.69 (0.59; 0.8)^a^	0.61 (0.53; 0.74)^a^	0.75 (0.65; 0.91)^a^	**<0.001**
DXA				
FM (kg)	33.62 (25.72; 44.6)^a^	37 (29.1; 46.3)^a^	31.8 (23.7; 40.6)^a^	**<0.05**
FM%	44.37 (40.49; 46.64)^a^	44.9 (42.2; 46.8)^a^	42.9 (39.7; 46)^a^	0.11
SMMa (kg)	19.19 (14.17; 23.17)^a^	19.9 (16.2; 23.8)^a^	16.8 (13.7; 22.8)^a^	0.37
SMMa%	23.37 (22.03; 24.69)^a^	22.8 (21.5; 24.4)^a^	23.7 (22.7; 25.4)^a^	**<0.05**
MFR	0.53 (0.48; 0.61)^a^	0.51 (0.47; 0.58)^a^	0.57 (0.49; 0.65)^a^	0.05
Muscle strength				
Handgrip max	24 (16; 33)^a^	26 (17; 30)^a^	22 (14.8; 35.2)^a^	0.9
Physical performance				
6-min walk test distance	551.38 ( ± 60.61)^c^	538 ( ± 61.9)^b^	563 ( ± 57.7)^b^	**<0.05**
TUG	7.02 (6.13; 8.02)^a^	7.52 (6.2; 8.19)^a^	6.82(6.07; 7.72)^a^	=0.1

IOTF international child cut-offs correspond to the BMI cut-offs at 18 years (27 for obesity grade 1, 30 for obesity grade 2, and 35 for obesity grade 3–morbid obesity) (21).

BIA, bioelectrical impedance analysis; BMI, body mass index; DXA, dual X-ray absorptiometry; FM, fat mass; FM%, fat mass/weight; IOTF, International Obesity Taskforce; MFR, muscle-to-fat ratio; n, number; NA, not applicable; SMMa, appendicular muscle mass; SMMa%, SMMa/weight; TUG, timed up and go test; WHtR, waist-to-height ratio.

a Data are presented as median (lower quartile; upper quartile).

b Data are presented as mean ± SD.

c Data are presented as percentage.

The bold values are statistically significant.

### 3.2 Presence of sarcopenia according to different diagnostic criteria

According to the different criteria used to define sarcopenia, its presence ranged from 6.32% (co-occurrence of handgrip strength (≤10th centile) according to Dodds et al. (25) and SMMa% (≤2^nd^ centile) according to McCarthy et al. (23) measured by BIA) to 97.89% (MFR cut-off value according to McCarthy et al. (23) measured by both DXA and BIA) (**Table 2**).

**Table 2:** Presence of sarcopenia according to different diagnostic criteria.

Handgrip strength (≤10th centile) (25) and SMMa% (≤2nd centile) (23) measured by DXA	Handgrip strength (≤10th centile) (25) and SMMa% (≤9th centile) (23) measured by DXA	Handgrip strength (≤10^th^ centile) (25) and SMMa% (≤2nd centile) (23) measured by BIA	Handgrip strength (≤10th centile) (25) and SMMa% (≤9th centile) (23) measured by BIA	MFR measured by DXA Cut-off points: 1.25 for boys of all ages, 1.1 for girls between 5 and 10 years old, and 0.8 for girls between 10 and 18 years old (23)	MFR measured by BIA Cut-off points: 1.25 for boys of all ages, 1.1 for girls between 5 and 10 years, and 0.8 for girls between 10 and 18 years (23)
15.79% (*n* = 15)	17.89% (*n* = 17)	6.32% (*n* = 6)	12.63% (*n* = 12)	97.89% (*n* = 93)	97.89% (*n* = 93)

Data are presented as percentage.

BIA, bioelectrical impedance analysis; DXA, dual X-ray absorptiometry; MFR, muscle-to-fat ratio; n, number; SMM%, appendicular muscle mass/weight.

### 3.3 Anthropometrical measures, body composition, muscle strength, and physical performance by the presence of sarcopenia

The characteristics of the participants according to the presence of sarcopenia are presented in **Table 3**. As only an assessment of both muscle mass and muscle strength is in line with the EWGSOP2 consensus, a definition of sarcopenia including both muscle mass and strength was used in the latter part of this study. The authors decided to use SMM% values in the ≤9th centile as a cut-off point for further analysis to avoid inconsistency (since values in the ≤10th centile were used as a cut-off point for grip strength).

**Table 3:** Characteristics of the study population and anthropometric data according to the presence of sarcopenia.

	Sarcopenia present	Sarcopenia absent	*p*-value
*n*	17.89%^a^ (*n* = 17)	82.11%^a^ (*n* = 78)	NA
Age (years)	10.7 (9.25; 13.1)^b^	12.9 (10.9; 15.5)^b^	0.2
Male sex	41.18%^a^ (*n* = 7)	57.69%^a^ (*n* = 45)	0.33
BMI	29.7 (26.7; 31)^b^	30.3 (27.8; 34.4)^b^	0.2
BMI *z*-score	2.72 (2.52; 3.29)^b^	2.95 (2.6; 3.52)^b^	0.27
WHtR	0.61 ( ± 0.06)^c^	0.6 ( ± 0.06)^c^	0.53
IOTF	**(1)** 41.18%^a^ (*n* = 7)	**(1)** 32.05%^a^ (*n* = 25)	0.77
	**(2)** 29.41% (*n* = 5)	**(2)** 33.33% (*n* = 26)	
	**(3)** 29.41% (*n* = 5)	**(3)** 34.62% (*n* = 27)	

Sarcopenia was defined as a co-occurrence of weak handgrip strength (≤10th centile) according to Dodds et al. (25) and low SMM% (≤9th centile) according to McCarthy et al. (23) (measured by DXA). IOTF international child cut-offs correspond to the BMI cut-offs at 18 years (27 for obesity grade 1, 30 for obesity grade 2, and 35 for obesity grade 3–morbid obesity) (21).

BMI, body mass index; IOTF, International Obesity Taskforce; n, number; NA, not applicable; WHtR, waist-to-height ratio.

a Data are presented as percentage.

b Data are presented as median (lower quartile; upper quartile).

c Data are presented as mean ± SD.

Sarcopenia was defined as the co-occurrence of weak handgrip strength (≤10th centile) according to Dodds et al. (25) and low SMM% (≤9th centile) according to McCarthy et al. (23) (measured by DXA).

Of the children with sarcopenia, 41.18% were boys and 58.82% were girls. Individuals with and without sarcopenia did not differ significantly in terms of age, gender, BMI, BMI *z*-score, WhtR, and severity of obesity according to IOTF criteria.

Regarding body composition measurements derived from DXA and BIA, sarcopenic individuals had significantly lower SMMa measured by BIA (*p* < 0.01) and SMMa measured by DXA (*p* < 0.01). Both DXA- and BIA-derived FM% were higher in children and adolescents with sarcopenia, while both DXA- and BIA-measured SMMa% and MFR were lower in sarcopenic individuals; however, the differences did not achieve statistical significance (**Table 4**).

**Table 4:** Body composition by the presence of sarcopenia.

	Sarcopenia present	Sarcopenia absent	*p*-value
BIA			
FM (kg)	23.5 (18.6; 33.7)^a^	30.8 (22.9; 40.8)^a^	0.11
FM%	38.9 ( ± 5.3)^b^	38.5 ( ± 6.36)^b^	0.85
SMMa (kg)	16.9 (13.9; 19.5)^a^	21.8 (16.8; 26.1)^a^	**<0.01**
SMMa%	25.5 ( ± 1.85)^b^	26.7 ( ± 2.7)^b^	0.09
MFR	0.66 (0.58; 0.77)^a^	0.7 (0.59; 0.82)^a^	0.4
DXA			
FM (kg)	30.3 ( ± 11.1)^b^	35.8 ( ± 13.1)^b^	0.11
FM %	45.2 (42; 47.1)^a^	44.1 (40.2; 45.9)^a^	0.38
SMMa (kg)	14.3 (11.6; 18)^a^	19.9 (14.8; 23.8)^a^	**<0.01**
SMMa %	22.6 (21.6; 23.7)^a^	23.5 (22.1; 24.8)^a^	0.07
MFR	0.5 (0.47; 0.58)^a^	0.53 (0.48; 0.62)^a^	0.24

Sarcopenia was defined as co-occurrence of weak handgrip strength (≤10th centile) according to Dodds et al. (25) and low SMM% (≤9th centile) according to McCarthy et al. (23) (measured by DXA).

BIA, bioelectrical impedance analysis; DXA, dual X-ray absorptiometry; FM, fat mass; FM%, FM/weight; MFR, muscle-to-fat ratio; SMMa, appendicular muscle mass; SMMa%, SMMa/weight.

a Data are presented as median (lower quartile; upper quartile).

b Data are presented as mean ± SD.

The bold values are statistically significant.

In comparison with individuals without sarcopenia, those with sarcopenia had significantly lower maximal handgrip strength and lower physical performance—a shorter distance of 6MWT and a longer time of TUG (**Table 5**).

**Table 5:** Muscle strength and physical performance in the presence of sarcopenia.

	Sarcopenia present	Sarcopenia absent	*p*-value
Muscle strength			
Handgrip max	12 (10.9; 16)^a^	27 (20; 35.8)^a^	**<0.001**
Physical performance			
6-min walk test distance	510 ( ± 69.5)^b^	560 ( ± 55.1)^b^	**<0.01**
TUG	8.22 ( ± 2.02)^b^	6.96 ( ± 1.4)^b^	**<0.05**

a Data are presented as median (lower quartile; upper quartile).

b Data are presented as mean ± SD.

The bold values are statistically significant.

### 3.4 Correlations between body composition, anthropometrics, muscle strength, and physical performance according to gender and age

Maximal handgrip was positively correlated with SMMa (kg) and SMMa% derived from both DXA and BIA, and MFR measured by BIA. Moreover, maximal handgrip was negatively correlated with WHtR. Distance of 6MWT correlated positively with BIA-measured SMMa% and BIA-MFR. In addition, 6MWT distance correlated negatively with BIA-FM% and BMI *z*-score. TUG was positively correlated with BIA-FM%, BMI *z*-score, WHtR, and IOTF categories and negatively correlated with BIA-SMMa% and BIA-MFR (**Table 6**).

**Table 6:** Correlations between anthropometrics, body composition, muscle strength, and physical performance according to gender and age.

	Handgrip max		6-min walk test distance		TUG	
	*r*	*p*-value	*r*	*p*-value	*r*	*p*-value
BMI *z*-score	0.05	0.63	**−0.33**	**<0.01**	**0.29**	**<0.01**
WHtR	**−0.27**	**<0.01**	−0.02	0.87	**0.29**	**<0.01**
IOTF	0.08	0.29	−0.12	0.09	**0.15**	**<0.05**
BIA						
FM%	−0.18	0.08	**−0.31**	**<0.01**	**0.22**	**<0.05**
SMMa (kg)	**0.46**	**<0.001**	0.04	0.68	0.06	0.59
SMMa%	**0.3**	**<0.01**	**0.34**	**<0.001**	**−0.22**	**<0.05**
MFR	**0.25**	**<0.05**	**0.31**	**<0.01**	**−0.22**	**<0.05**
DXA						
FM%	−0.12	0.24	−0.07	0.52	0.12	0.27
SMMa (kg)	**0.52**	**<0.001**	0.08	0.45	0.01	0.96
SMMa%	**0.22**	**<0.05**	0.19	0.06	−0.19	0.08
MFR	0.06	0.59	0.02	0.81	−0.12	0.27

IOTF international child cut-offs correspond to the BMI cut-offs at 18 years (27 for obesity grade 1, 30 for obesity grade 2, and 35 for obesity grade 3–morbid obesity) (21).

BIA, bioelectrical impedance analysis; BMI, body mass index; DXA, dual X-ray absorptiometry; FM%, fat mass/weight; IOTF, International Obesity Taskforce; MFR, muscle-to-fat ratio; SMMa, appendicular muscle mass; SMMa%, SMMa/weight; TUG, timed up and go test; WHtR, waist-to-height ratio.

The bold values are statistically significant.

## 4 Discussion

This study investigated the presence of SO according to different diagnostic criteria and evaluated components of sarcopenia (muscle mass, muscle strength, and physical performance) in obese children and adolescents. To our knowledge, it is one of the first studies that assessed all of the pediatric population according to the EWGSOP2 consensus statement (8).

The presence of SO in our study ranged from 6.32% to 97.89%, depending on the criteria used to diagnose sarcopenia. In our study, the presence of sarcopenia according to the MFR cut-off values introduced by McCarthy et al. (23) was 97.89% for both BIA- and DXA-derived measurements. In the study by Sack et al. regarding obese children and adolescents, the prevalence of sarcopenia according to the MFR cut-off value introduced by McCarthy et al. (23) was much lower (69.7%). In this study, BIA was used to evaluate body composition (27). In the study by Videira-Silva et al. regarding overweight adolescents, in which sarcopenia was defined as SMM% ≤p25 according to the youth reference charts introduced by McCarthy et al. (23) (measurements derived from BIA), the overall prevalence of sarcopenia was 26.9% (15). In our study, SMM% (derived from BIA) was also assessed according to the youth reference charts introduced by McCarthy et al. (23); however, the diagnosis of sarcopenia was based on both low SMM% (≤2nd centile or ≤9th centile) and weak handgrip strength according to Dodds et al. (≤10th centile) (25); therefore, the presence of sarcopenia was lower (6.32% for the cut-off value ≤2nd centile SMM% and 12.63% for the cut-off value ≤9th centile SMM%).

However, it should be noted that, to our knowledge, our study is the first to diagnose sarcopenia in obese children and adolescents with the use of muscle strength and muscle mass; therefore, the comparison with another study using the same diagnostic criteria is impossible. Only the assessment of both muscle strength and muscle mass is in line with the EWGSOP2 consensus; therefore, a definition of sarcopenia consisting of both muscle strength and mass was used in the further part of this study.

In our study, children with sarcopenia (defined as the co-occurrence of weak handgrip strength and low SMM%) had significantly lower SMMa measured by both BIA and DXA, lower maximal handgrip strength, and lower physical performance (shorter distance of 6MWT and longer time of TUG). Results regarding lower muscle strength and physical performance are in line with the study by Sack et al., in which individuals with sarcopenia had significantly lower jumping distance (standing long jump was used to evaluate muscle strength) and significantly lower cardiorespiratory fitness (used to determine physical performance) (27). The study by Videira-Silva et al. showed that in the group of adolescents classified as SMM% ≤p25, physical activity (number of minutes of physical activity during the week) was significantly lower than in the nonsarcopenic group (15).

Maximal handgrip was positively correlated with SMMa (kg) and SMMa% derived from both DXA and BIA, and MFR measured by BIA. Moreover, maximal handgrip was negatively correlated with WHtR. The study by Silva Neto et al., which included women from Brazil with a mean age of 64.92 ± 5.74 (range: 60–79), found a positive and significant correlation between handgrip strength and appendicular fat-free mass (AFFM; kg) measured by DXA (*r* = 0.41) (28). Physical performance measured by 6MWT and TUG correlated significantly with FM% assessed by BIA (negatively with 6MWT distance and positively with TUG), SMMa% assessed by BIA (positively with 6MWT distance and negatively with TUG), MFR measured by BIA (positively with 6MWT distance and negatively with TUG), and BMI *z*-score (negatively with 6MWT distance and positively with TUG). Moreover, TUG correlated positively with the WHtR and IOTF categories. These results are in line with the results of the study by Videira-Silva et al., in which physical activity (number of minutes of physical activity during the week) correlated positively with SMM% and MFR and negatively with FM% and BMI *z*-score (15).

According to the EWGSOP2 consensus on sarcopenia, numerous methods can be used in order to assess muscle mass quantity (8). Magnetic resonance imaging and computed tomography are regarded as gold standards for the noninvasive assessment of muscle quantity. However, these methods are associated with exposure to radiation, high cost, and lack of portability; therefore, they are not routinely used in muscle quantity assessment (29, 30). DXA is presently widely used among both clinicians and researchers since it provides a reproducible estimate of appendicular skeletal muscle mass in a few minutes when using the same instrument and cut-off points. Among the disadvantages of DXA are the lack of portability of the DXA instrument and the impact of the hydration status of the patient on DXA measurements (8). BIA is affordable, portable, and widely available; therefore, BIA device usage might be preferable to DXA (8). However, BIA-derived measurements are also influenced by hydration status of the patient, and it should also be noted that BIA does not measure muscle mass directly but estimates muscle mass with the use of conversion equation calibrated with a reference of DXA-measured lean mass in a specific population (8, 31). In our study, both DXA and BIA were used to evaluate muscle mass.

The lack of a consensus statement regarding diagnostic criteria for sarcopenia and sarcopenic obesity in children and adolescents hampers research in this field. The exact presence of SO in children and adolescents is unknown.

### 4.1 Study limitations

This study contains some limitations that should be mentioned. Firstly, the study sample did not represent the entire population of Poland since it only included participants from one clinic. Secondly, the number of participants included in the study was rather small. The pubertal status was not taken into account in this study because the Tanner stage was not recorded. Since puberty influences body composition and anthropometry, the lack of puberty status assessment could have an impact on the results.

Moreover, regarding the lack of a consensus statement concerning diagnostic criteria and cut-off points for SO in children and adolescents, the authors used various definitions in order to evaluate the presence of SO and introduced their own definition based on muscle mass and muscle strength—co-occurrence of weak handgrip strength according to Dodds et al. (25) and low SMM% according to McCarthy et al. (23). The definition is in line with the EWGSOP2 consensus statement (both muscle strength and muscle mass must be evaluated). Further studies, including larger and representative samples, are needed.

## 5 Conclusions

The presence of SO in our study ranged from 6.32% to 97.89%, depending on the criteria used to diagnose sarcopenia. In our study, children with sarcopenia (defined as the co-occurrence of weak handgrip strength and low SMM%) had significantly lower SMMa measured by both DXA and BIA, lower maximal handgrip strength, and lower physical performance. Maximal handgrip was positively correlated with SMMa (kg) and SMMa% derived from both DXA and BIA, and MFR measured by BIA. Moreover, maximal handgrip was negatively correlated with WHtR. The distance of 6MWT correlated positively with BIA-measured SMMa% and BIA-MFR. In addition, 6MWT distance correlated negatively with BIA-FM% and BMI *z*-score. TUG was positively correlated with BIA-FM%, BMI z-score, WHtR, and IOTF categories and negatively correlated with BIA-SMMa% and BIA-MFR. The lack of a consensus statement regarding SO diagnosis, diagnostic methods, and gender-, age- and race-specific cut-off points in the pediatric population hampers the progress of the research in this field.

## Data availability statement

Datasets include participants’ identifiable data. Requests to access the datasets should be directed to marcela.zembura@gmail.com.

## Ethics statement

The studies involving humans were approved by Medical University of Silesia bioethical committee. The studies were conducted in accordance with the local legislation and institutional requirements. Written informed consent for participation in this study was provided by the participants’ legal guardians/next of kin.

## Author contributions

MZ, PM, KC-B, PD, and PTD contributed to the conception and design of the study. MZ and KC-B organized the database. MZ performed the statistical analysis. MZ and PM wrote the first draft of the manuscript. MZ, PM, KC-B, PD, and PTD wrote sections of the manuscript. All authors contributed to the article and approved the submitted version.
